# What programs the size of animal cells?

**DOI:** 10.3389/fcell.2022.949382

**Published:** 2022-11-01

**Authors:** Shixuan Liu, Ceryl Tan, Mike Tyers, Anders Zetterberg, Ran Kafri

**Affiliations:** ^1^ Department of Molecular Genetics, University of Toronto, Toronto, ON, Canada; ^2^ Program in Cell Biology, The Hospital for Sick Children, Toronto, ON, Canada; ^3^ Department of Chemical and Systems Biology, Stanford University, Stanford, CA, United States; ^4^ Institute for Research in Immunology and Cancer, University of Montréal, Montréal, QC, Canada; ^5^ Department of Oncology-Pathology, Karolinska Institutet, Stockholm, Sweden

**Keywords:** cell size, cell size checkpoint, cell cycle, cell size homeostasis, cell growth, target size, cell size sensing

## Abstract

The human body is programmed with definite quantities, magnitudes, and proportions. At the microscopic level, such definite sizes manifest in individual cells - different cell types are characterized by distinct cell sizes whereas cells of the same type are highly uniform in size. How do cells in a population maintain uniformity in cell size, and how are changes in target size programmed? A convergence of recent and historical studies suggest - just as a thermostat maintains room temperature - the size of proliferating animal cells is similarly maintained by homeostatic mechanisms. In this review, we first summarize old and new literature on the existence of cell size checkpoints, then discuss additional advances in the study of size homeostasis that involve feedback regulation of cellular growth rate. We further discuss recent progress on the molecules that underlie cell size checkpoints and mechanisms that specify target size setpoints. Lastly, we discuss a less-well explored teleological question: why does cell size matter and what is the functional importance of cell size control?

## Introduction

**FIGURE 1:**
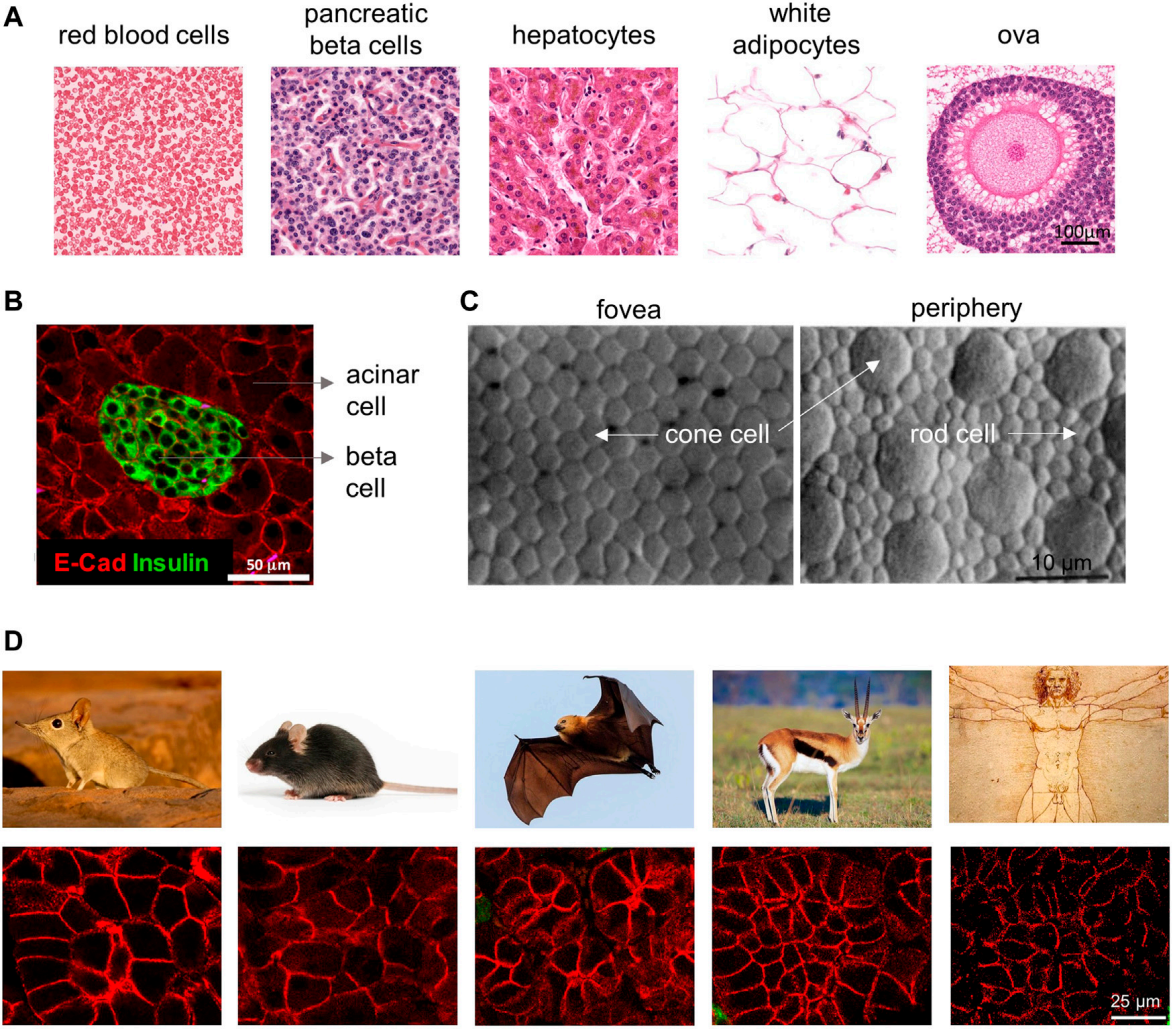
**Cell size differences among different cell types and species**. **(A)** In the human body, cells of different cell types can vary significantly in their size. Cell images are shown at the same magnification, adapted with permission from HistologyGuide.org. **(B)** Cell size differences in murine pancreatic cells. Note the pancreatic beta cells in the islet (insulin positive) have much smaller size than the surrounding acinar cells (insulin negative). Adapted from (Anzi et al., 2018). **(C)** Cell size in the retina not only differs by cell type (cone vs. rod) but also by the location in the tissue (fovea vs. periphery). Note that the cone cells are smaller in the fovea than ones in the periphery, which directly corresponds with the retina’s local visual resolution. Image is from (Curcio et al., 1990). **(D)** Pancreatic acinar cells vary in size in different mammalian species, from left to right are the Etruscan shrew, mouse, fruit bat, mountain gazelle, and human (tissue section images from Yuval Dor’s lab and animal images searched from Google).

Leonardo da Vinci’s *Vitruvian man* stands as a monument to the innate curiosity of humanity to uncover the code that reproducibly programs the quantities and proportions of life. Advances in modern biology have uncovered networks of gene regulation and signal transduction, yet it remains unknown how definite values and quantities are programmed and measured by these complex networks. Reproducible size differences over orders of magnitude distinguish animal species (Bonner, 2006). Within a given species, size differences characterize organs and cell types (Figure 1A). In the murine pancreas, for example, β cells are roughly half the size of their neighboring acinar cells (Figure 1B). These differences in cell size suggest that, during differentiation, different cell types are programmed with a specific target size (Figure 1C). At the molecular level, growth in cell size is often attributed to the conserved mTORC1 growth factor and nutrient sensing network (Fingar et al., 2002; Sabatini, 2017), but it remains unknown whether or how mTORC1 functions to specify a characteristic size for each of the many different cell types in the body.

Questions on cell size have been investigated for over a century since the start of modern cytology (Jorgensen and Tyers, 2004). Yet, molecular mechanisms that program the size of animal cells are only starting to be revealed. Until recently, a major focus of the field had been to resolve whether “active” homeostatic control of cell size exists for proliferating mammalian cells, i.e., whether cells actively monitor and adjust their cell size as opposed to size merely being a passive consequence of cell growth and division. Conversely, although less well-studied, size changes in non-proliferating cells are by definition the result of an active process that is independent of division. Reflecting on both old and recent literature, we present a convergence of evidence to suggest that individual cells actively maintain size homeostasis by regulating both the speed of cell cycle progression (i.e., cell cycle duration) and the rate of cell size growth (i.e., cellular growth rate). We further discuss recent advances on the molecular mechanisms underlying homeostatic control of cell size as well as target size specification. Last, we discuss the functional relevance of cell size and how cell size affects cellular, tissue and organismal level functions. While we mainly focus on proliferating mammalian cells, we also highlight examples of size control in terminally differentiated cells under physiological or pathological conditions for which mechanistic insights are less explored. We hope this review inspires more mechanistic and functional studies of cell size control in the future.

## The thermostat analogy of cell size control

**FIGURE 2:**
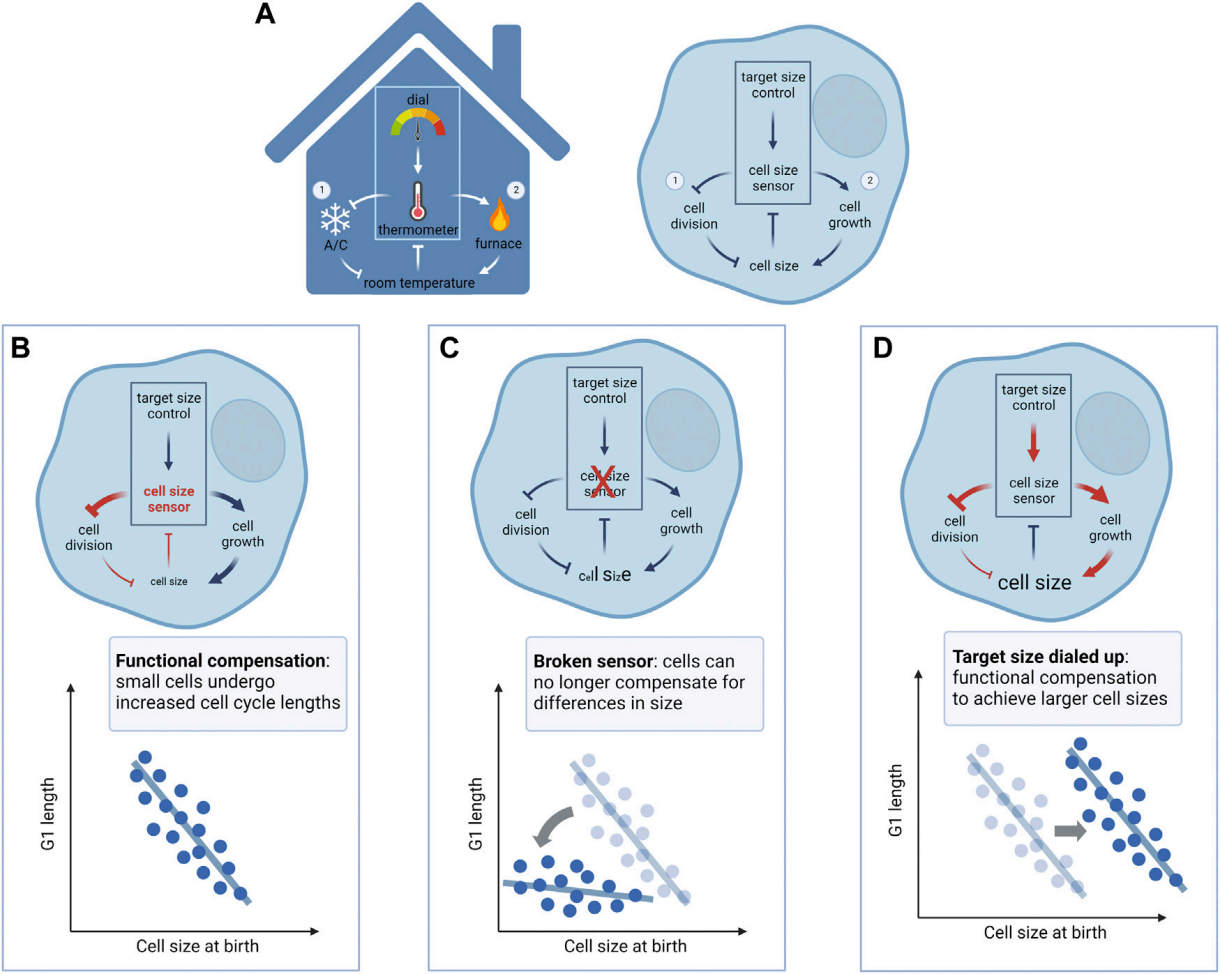
**A model for mammalian cell size control**. **(A)** Cell size regulation shares similar control circuits as a thermostat that controls the temperature of a room. Note that analogies between circuit components are based on the direction of regulation, i.e., faster cell division/cell cycle alone reduces cell size (turning on/up the air conditioner reduces room temperature), and faster cell growth alone increases cell size (turning on/up the furnace increases room temperature). Left panel: A thermometer measures the temperature of the room and compares it to the setpoint determined by the dial. If the room temperature is lower than the setpoint, the thermostat turns on the furnace and turns off the air conditioner to heat up the room. Conversely, if the room temperature is higher than the setpoint, the thermostat turns off the furnace and turns on the air conditioner to cool the room down. This dual-arm negative feedback regulation on the furnace and air conditioner maintains the room temperature at the setpoint. Right panel: Cell size control may involve a cell size sensor, relating a cell’s actual size with a “programmed” target size value, to regulate cell cycle progression (① cell size checkpoint) and cellular growth (② size-dependent regulation of growth rate), respectively. Cells that are smaller than their target size mainly compensate with a longer cell cycle mediated by size-dependent G1 extension, whereas cells that are larger than their target size mainly compensate with slowed cell growth, mediated by the upregulation of global protein degradation. This dual-arm negative feedback regulation on cell cycle and cellular growth maintains cell size relatively stable at the target size value. **(B)** With a properly functional size control mechanism (e.g., cell size checkpoint), small cells compensate with a longer period of growth in G1, allowing all cells to reach similar sizes at S phase entry. **(C)** When the homeostatic size control is perturbed (e.g., p38 inhibition, Rb1 knockout), cells would fail to compensate for their small size with a G1 extension, resulting in increased size heterogeneity. **(D)** When the target size is changed to a different level without perturbing the homeostatic control mechanism, cell size is shifted to the new setpoint while maintaining the compensatory G1 extension (e.g., CDK4 inhibition). Diagrams created using BioRender.com.

At an EMBO workshop on cell size regulation in 2016, Wallace Marshall suggested an interesting analogy for cell size regulation. Size control, according to Marshall’s analogy, may be compared to a thermostat in the maintenance of room temperature (Figure 2). A thermostat (cell size homeostasis) comprises a thermometer (cell size sensor) that senses the room temperature (current cell size) and adjusts the activity of the furnace and/or air conditioner (cell growth and cell cycle machineries, respectively) to reach and maintain room temperature at the setpoint level (target size). However, when one observes a change in the room temperature, this can result from perturbations to the thermometer, to the furnace/air conditioner, or to the set point temperature. Similarly, in an experiment where one observes a change in cell size, it may reflect: 1) perturbations to size sensing/homeostatic control (broken thermometer or thermostat circuit), 2) perturbations to the cell growth or cell cycle machinery (broken furnace/air conditioner), such as diminished mTORC1 signaling or a prolonged G1, or 3) a reprogrammed target size (thermostat dialed to a lower or higher set point temperature).

Although not a perfect comparison, we find this thermostat analogy helps to clarify certain conceptual ambiguities regarding cell size regulation and may help resolve some of the historical debates in the field (see next section). In particular, this model highlights the importance of distinguishing different types of cell size perturbations when interpreting experimental results. The analogy also offers a useful conceptual framework to dissect different aspects of cell size control.

## Historical and recent studies on the existence of the mammalian cell size checkpoint

### Emergence of the cell size checkpoint concept

The cell size checkpoint refers to a size control point in the cell cycle that restricts cell cycle progression of cells that are too small or too large. The term checkpoint is drawn from its original use by Hartwell and Weinert to describe control mechanisms that enforce dependency in the cell cycle (Hartwell and Weinert, 1989). By analogy to a thermostat, the cell size checkpoint represents not a single module but a part of the homeostasis circuit: the cell size checkpoint involves active size sensing (thermometer) and corresponding modulation of the cell cycle machinery (air conditioner) to maintain size at the setpoint level. In mammalian cells, the most prominent and well-studied size checkpoint exists at the G1/S transition, where we focus our discussion.

Compelling genetic experiments in both the fission yeast *Schizosaccharomyces pombe* and the budding yeast *Saccharomyces cerevisiae* established the existence of cell size checkpoints in single-celled organisms in the 1970s, either at the G1/S transition in budding yeast or the G2/M transition in fission yeast (Nurse, 1975; Fantes and Nurse, 1977; Johnston et al., 1977). In contrast, the literature on animal cell size control has posited conflicting models for decades (Conlon and Raff, 2003; Sveiczer et al., 2004; Lloyd, 2013). And yet, ironically, it was in animal cells that the cell size checkpoint was first discovered. In 1965, Zetterberg and Killander reported evidence suggesting that individual mouse fibroblasts are programmed with a definite size (Killander and Zetterberg, 1965a; 1965b). To quantify cell growth along the cell cycle, Zetterberg et al. developed a sophisticated experimental assay in which cells were photographed every 45 min for 30 h to track cell division. Then, cells were fixed, stained with Feulgen dye, and scanned by microinterferometry to measure cellular dry mass and by microspectrophotometry to measure DNA and RNA content. The live-cell tracking allowed the estimation of a cell’s division age, i.e., the time period since its last mitotic division. This setup produced the first reported single-celled joint measurements of cell size and cell cycle state. Analysis of the data revealed that variability in cell size decreases as cells transition from the G1 phase of cell cycle into S phase (Killander and Zetterberg, 1965a). Indeed, fibroblasts in early S phase are more similar in size than sister cells that just emerged from cell division.

In addition, Zetterberg et al. reported evidence for cell size-dependent regulation of G1 length (Killander and Zetterberg, 1965b). When comparing cell size and cell cycle duration of cells cultured on different slides, they noticed that populations with smaller average sizes at birth grow for longer periods in G1 and accumulated more mass before G1 exit so that all populations enter S phase at a similar size (Figure 2B). This discovery was later supported by other groups working on different cell types (Shields et al., 1978; Darzynkiewicz et al., 1979; Gao and Raff, 1997). However, these observations remained at the population level, as it was technologically impractical to continuously track cell size in live, irregularly-shaped animal cells. In the 1970s, size-dependent control of cell cycle duration was corroborated at single cell resolution in budding yeast (Hartwell et al., 1974; Johnston et al., 1977) and fission yeast (Nurse, 1975; Fantes and Nurse, 1977). Specifically, small cells compensate with longer periods of growth whereas large cells commit to division with little additional growth. These observations supported the notion of a “cell size checkpoint” (Rupes, 2002; Wood and Nurse, 2015). Genetic screens ensued in search for yeast size mutants, which notably yielded the genes *wee1* (Nurse and Thuriaux, 1980), *WHI1* (later renamed *CLN3*) (Sudbery et al., 1980; Nash et al., 1988), and *WHI5* (Jorgensen et al., 2002), all of which were named after the loss- or gain-of-function small size phenotypes. These genetic insights helped lay the foundation for understanding the molecular control of the cell cycle. While Zetterberg’s early work suggested a similar cell size checkpoint at the G1/S transition for animal cells, the molecular basis for this checkpoint remained enigmatic. Moreover, in both yeast and mammalian cells, it remains unsettled whether homeostatic size control involves only a single “point” (or a short period of the cell cycle) or is continuously adjusted over an extended cell cycle period (Garmendia-Torres et al., 2018; Liu et al., 2022). In this review, we define the cell size checkpoint in a broad sense wherein cell cycle progression is regulated in accordance with cell size to maintain size homeostasis.

### Historical debates on mammalian cell size checkpoints

Although the pioneering work by Zetterberg et al. presented compelling evidence for a cell size checkpoint that gates the G1/S transition according to cell size, the existence of a mammalian cell size checkpoint remained controversial for many decades (Wells, 2002; Lloyd, 2013). Initially, opposition to the cell size checkpoint (or a cell-intrinsic size control) was motivated by findings showing that cell growth in size and cell cycle progression can be independently influenced by separate types of growth factors (Zetterberg et al., 1984; Conlon et al., 2001; Echave et al., 2007). For example, under certain conditions (e.g., dilution of growth factors), many cell types such as rat Schwann cells show a reduced cell size without triggering an immediate delay in cell cycle entry. However, it is ambiguous as to whether these experimental conditions may have altered the cell’s target size. Following the thermostat analogy, if dilution of growth factors changes the cells’ target size (e.g., lowering the room’s setpoint temperature), one should not expect that a decrease in cell size (e.g., sudden drop of room temperature) would trigger a subsequent cell cycle extension (e.g., shutting down the air conditioner) before the cells reach a new homeostasis. Therefore, we reason that these experiments were not sufficient to either support or refute the cell size checkpoint, i.e., cell-intrinsic size control.

The controversy on the existence of a cell size checkpoint was further fueled by conflicting observations on whether conditions that shift cell size correlate with an *immediate* lengthening/shortening of cell cycle. For example, Conlon and Raff reported that Schwann cells shifted from serum-free to serum-containing medium required more than one cell cycle to equilibrate to their new, larger cell size (Conlon and Raff, 2003). In contrast, Dolznig et al. switched erythroblasts between two conditions that associate with different sizes, a smaller size when under physiological cytokines and a larger size when under constitutively active oncogene expression (Dolznig et al., 2004). This study revealed that erythroblasts immediately adapt in cell cycle length and protein synthesis rates following the switch, resulting in reversible changes in cell size between the two states that were achieved within approximately one cell cycle. The discrepancy in the speed of cell size adaptation may be explained by differences in the stringency of the cell size checkpoint in different cell types or states. A cell type with a stringent size checkpoint should exhibit a faster size adaptation. On the other hand, a cell type with a permissive size checkpoint might allow cells within a wide range of sizes to enter the next cell cycle phase and may take multiple cycles to reach the new size homeostasis. Related to this notion, a series of recent studies have investigated sizer, adder, and timer models of cell size control (Varsano et al., 2017; Cadart et al., 2018; Xie and Skotheim, 2020), originally proposed to explain cell size control in bacteria (Taheri-Araghi et al., 2015; Wallden et al., 2016). The sizer model assumes a strict cell size checkpoint requiring cells to reach an exact size value, such that all cells should end up with identical sizes within one cell cycle. The adder model, however, assumes that no specific target size is required to pass through the cell cycle. Instead, all cells, no matter large or small, accumulate the same amount of growth per cell cycle and therefore require multiple cell cycles to adapt to a new target size. Finally, the timer model assumes that cells grow for a constant period of time, independent of their starting sizes, and thus lack an inherent compensation mechanism. These overly simplified models describe only limited cases and recent studies suggest that size control is more complex (see below).

In addition, the debate on whether mammalian cell growth follows linear or exponential kinetics (Conlon and Raff, 2003; Mitchison, 2003; Sveiczer et al., 2004) also contributed to the controversy regarding the existence of a mammalian cell size checkpoint. This debate was fueled by mathematical calculations showing that stable distributions of cell size are inconsistent with exponential growth (Brooks, 1981; Tyson and Hannsgen, 1985) unless a size checkpoint is invoked. To many, these calculations suggested that if growth is linear, size checkpoints may not be necessary (Lloyd, 2013). Recently, with the development of better experimental methods for measurements of growth dynamics, actual kinetics of cell size growth were found to follow a pattern that is more complex than the simple linear or exponential models initially proposed (see below).

### Modern technologies provide further evidence for mammalian cell size checkpoints

In the past decade, new technologies have enabled high-precision and/or high-throughput measurements of cell size, including live-cell tracking of cell growth at single-cell resolution across an entire cell cycle. With these technical advances, a plethora of new data supports the conclusion that individual animal cells do indeed employ cell-intrinsic control to maintain size homeostasis.

Ginzberg et al. (2018) repeated Killander and Zetterberg’s time-lapse experimental design in human epithelial cells, measuring cell size and cell cycle stage by single-cell fluorescence microscopy and automated image processing. These modern techniques allowed quantification at a higher throughput and reaffirmed Zetterberg’s discovery that cell size variability narrows at S phase entry. Similarly, live-cell tracking of cell size by quantitative phase microscopy revealed that the coefficient of variation of cell dry mass decreases around S phase entry (Liu et al., 2022). Varsano et al. (2017) devised an experimental system that forces animal cells to grow in narrow channels. By constraining cells in an elongated cylindrical geometry, the authors effectively reduced the cell’s three-dimensional volume to a single length scale. Observations obtained with this experimental system are in favour of cell size checkpoints, showing that smaller cells have extended periods of growth in G1. This work also examined channels with different geometry and found that all cells grow to a similar size regardless of the channel geometry. This surprising result suggests that intrinsic cell size control can be independent of cell shape and mechanical forces, at least in the basophil cell lines used in the study. Another study utilized a fluorescence exclusion method and tracked the dynamics of cell volume and cell cycle progression in individual cells (Cadart et al., 2018). Similarly, the authors found a negative correlation between cell size and G1 duration for cultured mammalian cells. In a series of additional studies, we and others measured growth of individual cells throughout the cell cycle, and consistently found that newborn cells with a smaller size compensate with longer periods of G1, giving rise to a size distribution that is stable over generations (D’Ario et al., 2021; Ginzberg et al., 2018; Liu et al., 2018; Liu et al., 2022; Zatulovskiy et al., 2020).

### Cell size checkpoints *in vivo* and in exceptional cases

The evidence described above was mostly derived from measurements on cultured proliferating cells, which brings to question whether such size checkpoints are also implemented by cells *in vivo* (Lloyd, 2013). In certain exceptional situations, cell cycle and cell growth are decoupled. For example, during early embryogenesis before access to external nutrients, cleavage cycles entail cell division without growth. However, such physiological contexts likely represent a specific adaptation to achieve unique biological ends than the general rule. For example, the embryonic cell cycle lacks G1 and G2 phases and only consists of S phase and mitosis such that cell cycle checkpoints are absent (Finkielstein et al., 2001). It is reasonable to suspect that during later phases of development (e.g., post-implantation embryogenesis and postnatal growth), tissue renewal, and regeneration, cell size checkpoints possibly function to maintain cell size uniformity that is common in many healthy tissues. Indeed, careful measurements of cell size and cell cycle progression in plant meristem cells (Jones et al., 2017; Serrano-Mislata et al., 2015) and mouse skin epidermis (Xie and Skotheim, 2020) reported similar cell size checkpoints *in vivo*. To date, such single-cell measurements of cell growth and division *in vivo* are still sparse in the literature. Future research should examine additional cell types and/or species to test the generality of the cell size checkpoint. In addition, it is also worth examining whether the cell size checkpoint also functions in endoreplication cycles that generate polyploid cells such as hepatocytes, pancreatic acinar cells, trophoblasts, and osteoclasts (Edgar and Orr-Weaver, 2001; Anzi et al., 2018). Endocycles use similar G1/S regulatory machinery as mitotic cycles (Edgar and Orr-Weaver, 2001). Therefore, the G1/S size checkpoint may exist in endocycles to maintain stable cellular content per genome to ensure balanced transcriptional and translational activities (Marguerat and Bähler, 2012; Neurohr et al., 2019; Mu et al., 2020).

## Cell size homeostasis is further regulated by a cell-autonomous feedback control of growth rate

### Evidence for size-dependent regulation of cellular growth rate

In proliferating cells, a cell’s size (s) is the integral of the cellular growth rate (ν) over the duration of the cell cycle (*t*), i.e., $s=\underset{t}{\int }v\cdot dt$ . In addition to the size-dependent modulation of cell cycle length that is characteristic of cell size checkpoints, recent work suggests that the control of size homeostasis also involves cell-autonomous feedback control of cellular growth rate, i.e., the rate of volume or mass change (Figure 2A). Following the thermostat analogy, a thermometer (cell size hemostatic control) can employ parallel circuits involving both a furnace (cell growth rate regulation) and an air conditioner (cell cycle machinery) to reach and maintain homeostasis. Kafri et al. developed a novel analytic method, termed ergodic rate analysis (ERA), to extract dynamics of cell size growth from fixed epithelial cell populations for differently sized cells along a pseudotime cell cycle trajectory (Kafri et al., 2013). This analysis revealed that the estimated cell growth rate (rate of mass accumulation) is negatively correlated with size before the G1/S transition, possibly contributing to the decrease in cell size variability occurring concomitantly. In addition, Son et al. (2012) used a suspended microfluidic resonator (SMR) to quantify the instantaneous growth rates of individual mouse lymphoblasts with a precision of ∼3%. This study provided the first direct, high-precision measurements of single cell growth curves and revealed a decrease in growth rate variability at the G1/S transition. Following this work, two studies used different approaches to measure cellular growth rate in adherent mammalian cells of different sizes across the cell cycle (Cadart et al., 2018; Ginzberg et al., 2018). Ginzberg et al. (2018) estimated cell size from microscopy images of nuclear area and cell mass (total protein content), and Cadart et al. (2018) estimated cell size from single cell measurements on cell volume. Despite the differences in experimental methods, these two studies converged to conclude that individual cells modulate not only growth duration (e.g., G1 length) but also growth rate to maintain size homeostasis. These findings were also supported by recent work that measured dynamics of cellular dry mass by quantitative phase microscopy (Liu et al., 2022). Ginzberg et al. and Cadart et al. further independently showed that genetic and/or pharmacological perturbations to cell cycle duration triggered reciprocal and compensatory adjustments in cellular growth rate. In cultured RPE1 cells, Ginzberg et al. (2018) demonstrated that doxycycline-induced expression of cyclin E or p27 results in shorter or longer periods of growth in G1, respectively. Yet, these changes in growth duration are compensated by reciprocal changes in growth rate, such that cell size remains relatively constant. Cadart et al. (2018) showed that when treated with the CDK inhibitor Roscovitine, larger HeLa cells at birth compensate with slower rates of growth.

It is worth noting that the regulation on growth rate likely differs by cell types and cell cycle stages (Liu et al., 2022). For example, measurements on cellular growth by SMR found that cultured lymphocytes exhibit exponential growth kinetics where cell size positively scales with growth rate in a cell cycle-dependent manner (Son et al., 2012; Mu et al., 2020). There is also substantial literature that reported linear growth kinetics for size in certain cell types, but it has been controversial whether the measurements were accurate enough and/or correctly interpreted to make the claim (Conlon and Raff, 2003; Mitchison, 2003; Cooper, 2004, 2006; Sveiczer et al., 2004). To date, it remains a non-trivial task to generate high-quality measurements of growth dynamics in single mammalian cells. SMR, for example, is only compatible with suspended cells, whereas growth rate derived from time-lapse size measurements of nuclear size, cell volume (e.g., by fluorescence exclusion), or cell mass (quantitative phase microscopy) is very noisy at the single cell level and requires averaging across a large population to see a trend. Nevertheless, studies using these techniques have revealed interesting patterns of growth rate regulation and the underlying molecular mechanisms are starting to be revealed.

### Active proteolysis underpins size-dependent regulation of cellular growth rate

What is the mechanism of such size-dependent regulation of cellular growth rate? Recent evidence suggests that the underlying driver unexpectedly involves proteasome-mediated global protein degradation, rather than protein synthesis (Liu et al., 2021). This study compared global rates of protein synthesis and degradation in differently sized cells across cell cycle stages in both unperturbed conditions or conditions that trigger a size-dependent compensation in cellular growth. These experiments demonstrated that the rate of protein synthesis scales linearly with cell size whereas the rate of protein degradation scales superlinearly, suggesting an activation of protein degradation pathways in large cells. For example, reducing the activity of cyclinE/CDK2 extends the cell cycle and subsequently triggers a compensatory slowdown of cellular growth, which involves upregulated proteasome-mediated global protein degradation. This finding suggests that the growth rate regulation in G1 mainly involves control of protein degradation, which was further supported by an independent study by Liu et al. (2022). Interestingly, it was found that large cells at the G1/S transition demonstrate hyperactivated protein degradation, even when compared to similarly-sized or larger cells at S or G2 phases (Liu et al., 2021). Taken together, these new discoveries suggest that the homeostatic size control at G1/S transition involves both the cell size checkpoint and protein degradation-mediated growth rate regulation.

## Molecular control of cell size checkpoints and the hunt for cell size sensors

In the past decade, the field has started to discern the molecular mechanisms underlying the cell size checkpoint and cell size sensing. Studies on different systems yielded different models for cell size sensing. Systematic screens on cell size have revealed many critical genes and proteins involved in size regulation in different yeast species (Jorgensen et al., 2002; Zhang et al., 2002; Navarro and Nurse, 2012; Soifer and Barkai, 2014; Sellam et al., 2019) as well as mammalian cells (Liu et al., 2018). Yet, it remains unsettled whether a single molecule or pathway functions as a ruler to measure cell size, and whether different species and/or cell types share a conserved mechanism or have evolved distinct adaptive mechanisms for size checkpoint and sensing. Below we discuss progress made in different systems.

### Budding and fission yeast

These two yeast model systems have pioneered the discovery of genes that activate or inhibit cell division, typically manifest as large or small sized cells in loss-of-function strains, respectively. As a primary countervailing force that balances growth, division may be triggered by the accumulation of cell cycle activators or the dilution of cell cycle inhibitors. Evidence exists for both mechanisms in both yeasts.

In the fission yeast *S. pombe*, size-dependent expression of phosphatase Cdc25, which activates the cyclin-dependent kinase Cdc2 to initiate mitosis, appears to link cell size at division (Keifenheim et al., 2017). Conversely, a potential cell size sensing mechanism based on inhibitor dilution involves the spatially restricted proteins Pom1 and Cdr2 (Martin and Berthelot-Grosjean, 2009; Moseley et al., 2009). Cdr2 is medially bound and promotes mitotic entry, whereas Pom1 localizes at the two poles and inhibits Cdr2 when cells are small. As cells elongate, Pom1 concentration at the medial zone drops, releasing cells from Pom1 inhibition, triggering mitotic entry. Follow-up work from both groups later found that Pom1 levels at the cell median was constant regardless of cell length, and that deletion mutants of Pom1 and Cdr2 nevertheless retain cell size homeostasis, suggesting the existence of additional or compensatory size-control mechanisms (Bhatia et al., 2014; Wood and Nurse, 2013). Interestingly, Facchetti et al. (2019) reported that *S. pombe* mutants of different widths divided at the same surface area as control, but at different lengths or volumes, suggesting that cells may directly sense cell surface area or surface-to-volume ratio rather than cell size.

In the budding yeast *S. cerevisiae*, the G1 cyclin Cln3 and the G1/S transcriptional inhibitor Whi5 were identified as strong size regulators from genetic screens (Nash et al., 1988; Jorgensen et al., 2002). Cln3 has long been implicated as a critical upstream activator of cell division based on its potent dosage-dependent ability to initiate the G1/S transition and commitment to division, called Start in yeast (Nash et al., 1988; Tyers et al., 1993). The Cln3-Cdc28 (Cdk1) kinase is thought to phosphorylate both Whi5 and the SBF transcription factor complex in late G1 phase, thereby alleviating Whi5 repression and activating G1/S transcription (Costanzo et al., 2004; de Bruin et al., 2004; Wagner et al., 2009). More recently, an inhibitor dilution model has been proposed. Schmoller et al. (2015) reported that while other G1/S regulators appear to maintain a constant concentration as cells grow in G1 phase, Whi5 is subject to attenuated synthesis in G1 and is therefore diluted in concentration as cells grow in size. This dilution may trigger Start once the Whi5 concentration drops below a critical threshold in late G1 phase. However, other quantitative studies have reported that the concentration of Whi5 is independent of cell size and time, i.e., Whi5 continues to be synthesized throughout G1 phase in concert with cell growth (Dorsey et al., 2018; Litsios et al., 2019; Sommer et al., 2021; Litsios et al., 2022). Genetic evidence suggests that constitutive expression of Whi5 at physiological levels does not alter the coupling of size with passage through Start (Barber et al., 2020). In contrast to Whi5 dilution, recent single cell measurements suggest that a burst in global protein synthesis in late G1 phase results in the rapid accumulation of the highly unstable G1 cyclin Cln3, which then triggers Start and G1/S transcription by the phosphorylation-dependent inactivation of Whi5 (Litsios et al., 2019; Liu et al., 2015; Sommer et al., 2021). The Whi5 dilution model remains contentious as the critical underlying quantitative measurements depend heavily on appropriate controls for Whi5 signal loss (Litsios et al., 2022; Schmoller et al., 2022). Additional regulators and mechanisms may couple cell size, growth, and Start in budding yeast. For example, quantitative single cell analysis suggested that the SBF subunit Swi4 is initially limiting in early G1 phase with respect to its binding sites in G1/S promoters but accumulates as cells grow (Dorsey et al., 2018) and may titrate available binding sites as cells approach Start (Wang et al., 2009). Intriguingly, super-resolution microscopy studies suggest that Swi4 and other G1/S transcription factors form discrete clusters of fixed size that increase in number as cells grow throughout G1 phase (Black et al., 2020). In another example, the Aldea group uncovered a potential role for Ydj1, a chaperone regulating Cln3 degradation and localization (Ferrezuelo et al., 2012). Another recent study proposed that Cln3-Cdc28 may directly phosphorylate and activate RNA Poll II to initiate G1/S transcription (Kõivomägi et al., 2021). Work by Chen et al. (2020) suggests that size homeostasis in the budding yeast likely involves changes in the concentrations of multiple proteins. This study found that a set of cell cycle activators increase in concentration as cells grow in size while another set of cell cycle inhibitors tend to decrease in concentration, and that the countervailing effects of these positive and negative regulators determines the size of cells at S phase entry (Chen et al., 2020). Similar differential scaling of macromolecular concentrations and organelle content over cell size as a function of senescence has been recently reported in human cells (Cheng et al., 2021; Lanz et al., 2021). Beyond cell size regulation under homeostatic conditions, many studies have investigated how nutrient conditions alter cell size in yeast, and multiple factors have been implicated as nutrient sensors or effectors, including Cln3, Whi5, G1/S transcription factors, nutrient-sensing kinases, and metabolic status (Dorsey et al., 2018; Litsios et al., 2019; Qu et al., 2019; Sommer et al., 2021; Tollis et al., 2022). Despite the powerful molecular genetics of yeast models, it is clear that further quantitative experiments will be needed to discriminate between countervailing models and to understand how different signals are integrated to effect robust yet adaptive size homeostasis.

### Mammalian cells

To probe the factors underlying the cell size checkpoint in mammalian cells, Liu et al. (2018) employed a high-throughput screening approach to systematically search for perturbations that result in loss of cell size (total protein mass) control. Specifically, the authors looked for perturbations that increase cell size variability and also disrupt the function of the G1/S size checkpoint, resulting in the loss of G1 length compensation in small cells (Figure 2C). It was found that components of the p38 MAPK pathway were highly enriched among the top hits of the screen. In contrast, the mTOR pathway, a master regulator of cell growth, is not required for the proper function of the checkpoint, i.e., small cells under mTOR inhibition still compensate with longer G1. The work further demonstrated that small cells undergo p38 activation, which delays their G1/S transition. Inhibition of p38 MAPKs by either chemical inhibitors or genetic knockdown leads to the loss of the compensatory G1 extension in small cells, resulting in faster proliferation, smaller cell sizes, and increased size heterogeneity. It was later independently reported by Sellam et al. (2019) that Hog1, a yeast homolog of mammalian p38 MAPK, similarly underlies the G1/S cell size checkpoint in the opportunistic yeast pathogen *Candida albicans*. This result suggests that the p38 MAPK may be an evolutionarily conserved regulator of cell size across eukaryotes. However, it remains to be discovered how p38 MAPK or its upstream regulators biophysically ‘sense’ cell size. The p38 MAPK pathway is canonically viewed as a stress response pathway that is activated by multiple types of stress stimuli, including cytokines, heat shock, and osmotic shock; notably, the latter induces fast and often drastic changes in cell volume (Han et al., 2020). This suggests an interesting possibility that changes in cell mass and volume are sensed, at least in part, through a shared mechanism. It is also possible that deviations in size homeostasis activates stress sensing pathways, which then integrate to activate the p38 MAPK and inhibit cell cycle progression.

In parallel, inhibitor dilution mechanisms have been reported for Rb in mammalian cells (Zatulovskiy et al., 2020), and for KRP4, a plant-specific G1/S inhibitor, in plant shoot meristem cells (D’Ario et al., 2021). The p16-cyclin D-CDK4/6 axis that mediates the phosphorylation-dependent inactivation of the Rb tumor suppressor, which inhibits the E2F family of G1/S transcription factors, has long been implicated in cell cycle commitment and, by extension, size control in mammalian cells (Sherr, 1996). This conventional view of phosphorylation-mediated inactivation of Rb has been challenged recently, however, in that size-dependent G1/S transition may not require progressive phosphorylation of Rb by cyclin/CDKs but is instead driven by size-associated Rb dilution (Zatulovskiy et al., 2020). It was suggested that Rb phosphorylation possibly remains constant throughout most of G1 and that the G1/S transition may be initiated before Rb hyperphosphorylation occurs (Rubin et al., 2020; Zatulovskiy et al., 2020). Analogous to the proposed Whi5 dilution mechanism in yeast (Schmoller et al., 2015), Zatulovskiy et al. (2020) reported that the amount of Rb is maintained relatively constant in the G1 phase and should therefore be diluted as cells grow in size. Rb synthesis was found to be restricted to the S and G2 phases, and mother cells were observed to partition equal amounts of Rb to both daughter cells in a size-independent manner, but the specific mechanisms are unknown. Knocking out Rb results in smaller cell sizes, higher cell size variability, and disruption of the size-dependency in G1 duration, similarly to the previously reported p38 inhibition phenotype (Liu et al., 2018; Zatulovskiy et al., 2020). Given that the p38 MAPK pathway is upstream of Rb in the regulation of G1/S transition (Thornton and Rincon, 2009; Tomás-Loba et al., 2019), it is plausible that both proteins function in a shared pathway downstream of cell size sensing to regulate cell size-dependent S phase entry.

### Cells in tissues and of other systems

Past pursuits for cell size sensors concentrated on single-celled yeast or cultured cells. Do animal cells in an *in vivo* tissue context employ further size sensing mechanisms? Mechano-sensing is a likely candidate. A study by Boehlke et al. (2010) showed that kidney epithelial cells regulate their size in response to the flow of extracellular fluid (i.e., urine flow *in vivo*) through activation of the LKB1-mTORC1 pathway induced by the primary cilia. Additionally, cardiac muscles are known to undergo hypertrophy under excessive mechanical load from high blood pressure or volume overload (Pitoulis and Terracciano, 2020). Studies on mechanobiology have revealed that mammalian cells can sense mechanical forces and regulate the cell cycle accordingly. High local mechanical stress was found to inhibit cell proliferation through the Hippo-YAP pathway (Pan et al., 2016); conversely, exerting a stretching force promotes cell proliferation (Streichan et al., 2014). A recent study on growing epithelial cell monolayer sheets also found that mechanical tension more strongly predicts a cell’s G1 duration compared to the cell area and area growth rate, suggesting that cells may directly sense mechanical forces to determine the timing of G1/S transition (Uroz et al., 2018). In addition to its role in regulating the cell cycle, it is possible that mechanical forces also regulate cellular growth: pushing forces may inhibit cell growth and therefore alleviate local mechanical stresses, whereas stretching forces may promote cell growth to reduce the initial stretch. Such a biophysical negative feedback regulation between mechanical forces and cell size may be critical for cell size uniformity at the tissue level. Direct experimental evidence for this hypothesis is still lacking, but recent work from different groups suggest that the Hippo-YAP pathway may function as a mechanotransducer to regulate cell size and volume independent of its role in the cell cycle (Gonzalez et al., 2018; Perez-Gonzalez et al., 2019; Mugahid et al., 2020).

In addition, others have developed mathematical models on axon length sensing in neurons (Rishal et al., 2012; Perry et al., 2016) and flagellum length sensing in *Chlamydomonas reinhardtii* (Hendel et al., 2018). These models suggest that size sensing may be an emergent phenomenon of a biochemical network instead of relying on a single sensor molecule. Interestingly, a study using cell-free droplets of *Xenopus* egg extracts recapitulated chemical oscillations of the embryonic cell cycle in oil-encapsulated droplets and found that smaller droplets had longer cycle period, resembling that of yeast and animal cells (Guan et al., 2018). Another study on a non-cell-cycle transcription oscillator also observed that smaller droplets had longer cycle periods (Weitz et al., 2014). As these droplets were generated from the same extract preparation, they should have the same expected concentrations of different types of molecules. Why do smaller droplets have longer cycle periods? A possible explanation is that smaller droplets have higher stochasticity in the number of different types of encapsulated molecules (Box 1).

Box 1 **Why do smaller cell-free droplets have longer cycle periods?**

Consider the chemical oscillation (e.g., cell cycle) in the droplets as a biochemical reaction of *N* rate limiting steps, and that in each step, a gene is translated in a linear rate to reach a normalized threshold 1 to activate the next reaction. In the embryonic cell cycle, for example, it can be considered to have one rate limiting step of cyclin B translation. Assume that the translation rate has a normal distribution *X(u,v)*, where *u* represents the distribution mean and *v* the standard deviation. Then for any cell, the cell cycle duration is N*E(1/X), where *E* represents the expectation (mean). When the cell is large enough, the translation rate equals *E(X)=u*, and the cycle duration is *N/u*. When the cell is small, the average duration of the cell cycle is N*E(1/X). According to Jensen's inequality, N*E(1/X)≥N/E(X)=N/u.

## Mechanisms that determine target size

The cell size checkpoint contributes to the control of size homeostasis. However, the setpoint of such a checkpoint can be dynamically adjusted during cellular differentiation and under different environmental conditions, similar to how the setpoint of a thermostat can be dialed. What is the mechanism that programs target size? Indications for roles of the CDK4-cyclin D1/Rb pathway in cell size control have previously been identified. CDK4/cyclin D1 functions to promote cell growth independently of cell cycle progression in both *Drosophila* and *C. elegans* (Datar et al., 2000; Korzelius et al., 2011), while the size of isolated murine hepatocytes scales with the number of *Rb1* alleles (Zatulovskiy et al., 2020). Tan et al. (2021) recently identified that CDK4 is involved in the target size specification of mammalian cells. In previous work on cell size checkpoint (Liu et al., 2018), the same group developed an experimental assay that quantifies the level of compensation (i.e., G1 length extension) in small cells under different levels of mTORC1 inhibition. This assay revealed two types of cell size regulators, which were termed sensors and dials, following the thermostat analogy. Perturbing sensors, such as p38 MAPK, disrupts the G1 length compensation (i.e., small cells do not compensate with longer G1), implicating their role in the cell size checkpoint. In contrast, perturbing dials, such as CDK4, shifts cells to a different size without interfering with the G1 length compensation, indicating a reprogramming of target size (Figure 2D). Using the same assay, Tan et al. (2021) characterized hits from a previously published cell size screen (Liu et al., 2018). Among all hits analyzed, CDK4 inhibitors produced the biggest shift in size (up to ∼80%) while still maintaining the size-dependent checkpoint at G1/S transition. Moreover, the observed influence on cell size is dose-dependent, suggesting that CDK4 activity fine tunes target size in a continuous manner. Interestingly, knockdown of CDK2 or CDK6 also results in a dial phenotype but has a much smaller influence on cell size (∼10%–20%), highlighting a distinct role for CDK4 in target size specification.

The strong influence of CDK4 on cell size suggests that in addition to its role in cell cycle progression, CDK4 also regulates growth rate. Indeed, its cyclin partner, cyclin D, has been shown to be a key mediator of cell growth in both plant and animal cells (Cockcroft et al., 2000; Nelsen et al., 2003). Decreases in CDK4 activity are also associated with increased rates of protein synthesis, activities of growth-promoting pathways (e.g., mTORC1, c-Myc, ERK), and overall bioenergetic capacity (Tan et al., 2021). Previously, p38 activity was found to be selectively upregulated in cells that are smaller than a given size threshold (Liu et al., 2018). Tan et al. (2021) further found that this threshold is determined by CDK4 activity. While links between p38 and CDK4 have been identified (Casanovas et al., 2000; Thoms et al., 2007), it remains unexplored how these two signaling pathways are coupled to maintain cell size homeostasis at a given CDK4-determined target size. Interestingly, genetic knockdown of *Rb1*, a direct downstream target of CDK4, results in perturbations to the G1/S size checkpoint and homeostatic control of cell size (Zatulovskiy et al., 2020). These studies show different influences for CDK4 and Rb1 on cell size - while perturbations to CDK4 activity in RPE1 cells and primary human fibroblasts result in shifts in cell size with minimal influence on size homeostasis (Tan et al., 2021), perturbing Rb1 in mouse hepatocytes leads to an increase in size heterogeneity (Zatulovskiy et al., 2020). The differential size effects of CDK4 and Rb1 may be explained by their tissue- and cell type-specific growth effects (Lin et al., 1996; Datar et al., 2000). Alternatively, it may suggest that CDK4 regulates cell growth through both Rb1-dependent and Rb1-independent mechanisms, as hinted by previous studies in *Drosophila* (Datar et al., 2000; Emmerich et al., 2004), *C. elegans* (Korzelius et al., 2011), and mammalian cells (Lee et al., 2014).

It is important to note that perturbations to either the cell size checkpoint, target size specification mechanism, or cell cycle progression can result in significant changes in cell size, particularly when probed at a population-averaged level. It is well recognized in the field that one needs to delineate a gene’s influence on the cell cycle from that on cell size. This is because perturbations to the cell cycle may affect cells’ distribution across cell cycle stages. Therefore, cell size may have appeared different at the population average yet remained identical when comparing the same cell cycle stage. Just as how cell size relates to the cell cycle, the control of cell size homeostasis and target size specification are two separate but coupled processes. It is therefore necessary to delineate the two to identify specific factors that separately control each, ones that regulate both, and those that coordinate the two processes.

## Cell size and function: Why does cell size matter?

Despite much recent work on the mechanisms of size control, past research has skirted the key question of the biological significance of cell size (Lloyd, 2013; Björklund and Marguerat, 2017; Miettinen et al., 2017). Nevertheless, various studies have identified interesting links between cell size and animal physiology/pathology (see below). In humans, deregulation of cell size and morphology has been associated with various diseases such as breast and small cell lung cancers (Asadullah et al., 2021; Bell and Waizbard, 1986; Lee et al., 1992), hypoinsulinemia (Giordano et al., 1993; Pende et al., 2000), neurological disorders (Kwon et al., 2001, 2003), and aging (Buchwalter and Hetzer, 2017; Tiku et al., 2017; Neurohr et al., 2019). In this section, we discuss this fundamental question and present examples and hypotheses on how cell size may affect different cellular, tissue, and organismal functions.

### A structural requirement

**FIGURE 3:**
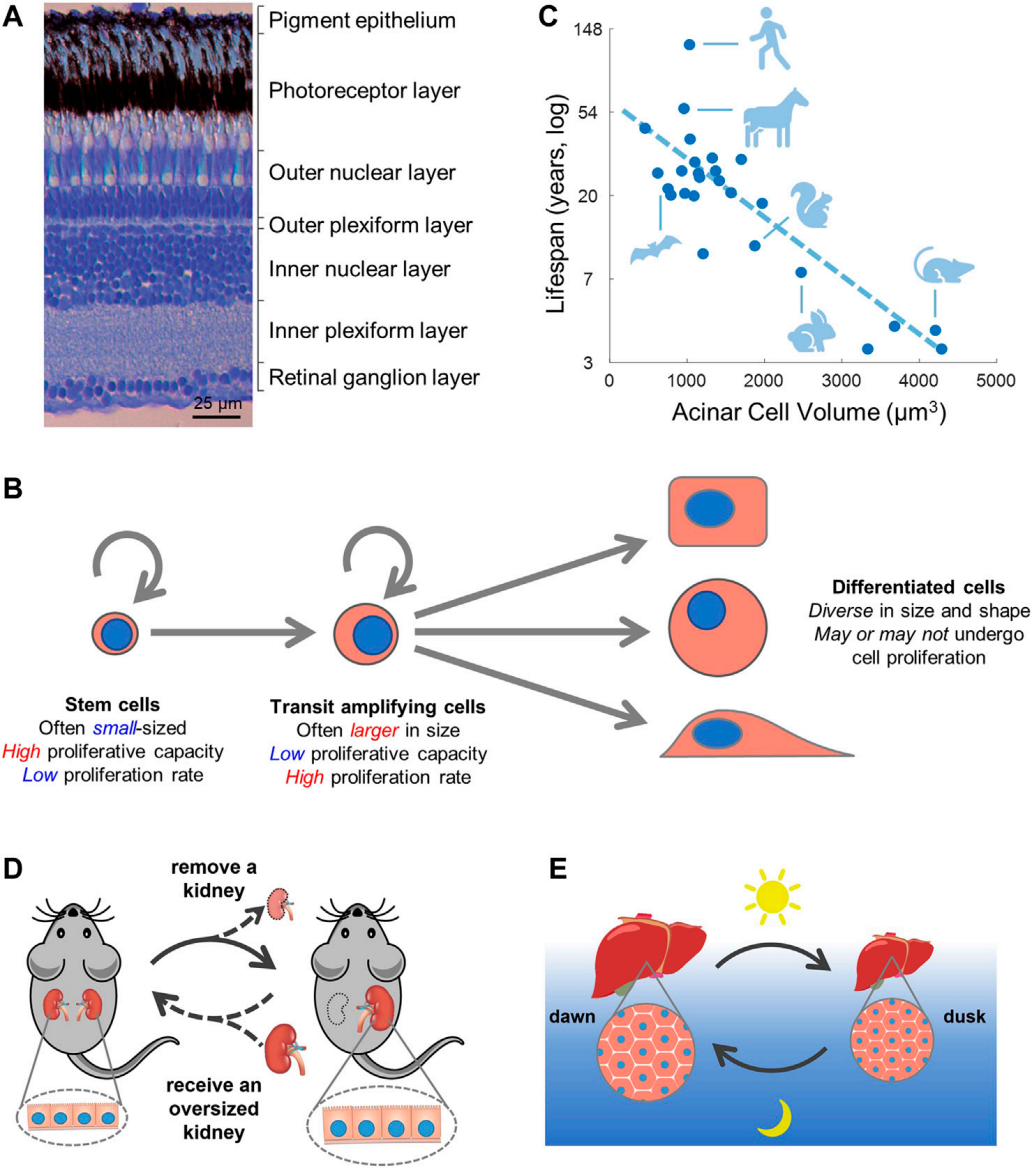
**Cell size and function**. **(A)** In the retina, cell size is tightly regulated to match the layered tissue structure. Image is from (Gramage et al., 2014). **(B,C)** Small cell size is correlated with stemness **(B)** and longer lifespan **(C)**. Panel **(C)** is adapted from (Anzi et al., 2018), which examined the size of pancreatic acinar cells among 24 mammalian species and identified a negative correlation between cell size and animal lifespan. **(D,E)** Cell size regulation as a general strategy to modulate organ size and function. **(D)** Animals removed of one kidney experience compensatory growth in the remaining kidney mainly through increases in cell size (Johnson and Vera Roman, 1966). Conversely, replacing a normal sized kidney with an oversized one induces a regression in kidney size (Churchill et al., 1991; Salvatierra et al., 1998), potentially through changes in cell size. **(E)** Murine hepatocytes oscillate in size from dawn to dusk following a circadian rhythm (Sinturel et al., 2017). Note that the mouse is nocturnal and eats at night.

Just as all the parts in a physical machine must precisely match in size for the machine to function properly, it is likely critical for cells in the body to coordinate in size with each other and with the tissue structure. For example, skeletal muscle fibers usually span the entire length of a muscle (Heidlauf and Röhrle, 2014) and many neurons also extend a long distance from the central nervous system to peripheral target tissues (Rich and Terman, 2018). The nervous system provides a particularly compelling example in which function depends on precise size control to match the tissue structure (Figure 3A).

Early research on cell size control in neurons found that neuronal size is regulated by extrinsic signals, such as nerve growth factors, secreted by peripheral targets that couple rates of protein synthesis with degradation (Purves et al., 1988; Franklin and Johnson, 1998). Such extrinsic size control allows neurons to adjust in size in accordance with its surrounding tissues during development and regeneration. In addition to extrinsic size control, several recent studies also proposed intrinsic mechanisms of axon length control (Lallemend et al., 2012; Rishal et al., 2012; Perry et al., 2016). Interestingly, a systematic analysis of dendrite morphology in cortical neurons revealed that the size of dendrites (length and number of branches) is cell-autonomously controlled, such that fluctuations in one region of a neuron are systematically counterbalanced by the remaining dendrites in the same cell (Samsonovich and Ascoli, 2006).

To maintain proper tissue structure, cells need to coordinate their size and shape but are not necessarily required to be at certain absolute sizes. In 1945, Gerhard Fankhauser published a fascinating paper showing an almost perfect maintenance of organ size and function in the salamander larvae despite significant changes in cell size (Fankhauser, 1945). In this study, haploid or polyploid animals that have smaller or larger cell sizes, respectively, maintained similar organ and body sizes as normal diploids by adjusting cell number and shape. While this seems to suggest that cell size does not necessarily impact overt physiological function, it raises the question of why cells in normal diploids have specific characteristic sizes. Resolution of this question remains an outstanding problem.

### An optimal cell size for growth and metabolism

While it is evident that certain cell types need to be of certain sizes, it is less obvious how cell size matters for others. One hypothesis is that there may exist an optimal size for particular functions. Supporting this notion, a study quantified mitochondrial activity across cells of different sizes and found that size-normalized mitochondrial level peaks at an intermediate cell size and drops in either large or small cells (Miettinen and Björklund, 2016). This observation suggests that an optimal cell size exists to maximize mitochondrial metabolism, which would be important for metabolically demanding processes such as cellular growth. Indeed, a similar trend has been observed for cellular growth rates. Although rates of cellular growth (in cell mass or volume) tend to increase with cell size (with the exception of G1/S transition), recent studies revealed a decline in growth rate and/or efficiency (size-normalized growth rate) in exceedingly large cells (Cadart et al., 2018; Ginzberg et al., 2018; Goranov et al., 2013; Liu et al., 2021; Neurohr et al., 2019; Son et al., 2012; Varsano et al., 2017), implying an optimal cell size range for tissues that undergo rapid growth. In microorganisms, optimal sizes may depend on nutrient conditions. For example, budding yeast show a reduced size upon nutrient limitation (Johnston et al., 1977). Interestingly, a multi-decade experiment that grew *E. coli* on rich media over 50,000 generations has found that cells consistently evolved larger sizes, suggesting higher fitness for large cells under rich nutrients (Grant et al., 2021).

What is the biophysical and biochemical basis for the existence of an optimal cell size? A well-known mechanism is that large cells have reduced surface area to volume ratio, which negatively impacts cross-membrane absorption and secretion (Miettinen and Björklund, 2017; Björklund, 2019). DNA content is also a potential limiting factor for cellular growth and function in large diploid cells. It was suggested that in both yeast and mammalian cells, a low DNA:cytoplasm ratio contributes to functional decline and senescence (Neurohr et al., 2019). Another possible mechanism is that the optimal function of a cell requires matched sizes and/or numbers of different intracellular molecules and structures, but these quantities may not always scale linearly with cell size. For example, many organelles have larger sizes in larger cells (Marshall, 2020), such as the nuclei, nucleoli, spindle, mitochondria, and endoplasmic reticulum. However, a recent quantification revealed that the protein content of different organelles scale with cell size at different slopes (Cheng et al., 2021), with the nucleus, lipid droplet, and mitochondrial outer membrane scaling sublinearly and the lysosome, autophagosomes, endoplasmic reticulum, and mitochondrial inner membrane and matrix scaling superlinearly with cell size. Together, these studies suggest that efficient growth is associated with an optimal size; the mechanisms that underlie this control and how various subcellular compartments contribute to this optimality remain to be investigated.

### Cell size, stemness, and lifespan

Stem and progenitor cells are critical for tissue homeostasis and regeneration. Interestingly, stem and progenitor cells are generally smaller in size compared to their differentiated progenies (Figure 3B) (Li et al., 2015; Lengefeld et al., 2021). For example, early studies using size elutriation have found that smaller epidermal keratinocytes and fibroblasts had higher proliferative capacity (Barrandon and Green, 1985; Angello et al., 1987). Smaller keratinocytes also show higher levels of the stem cell marker p63 (Parsa et al., 1999) and lower involucrin synthesis, an indicator of differentiation (Watt and Green, 1981). Hematopoietic stem cells that generate all blood lineages are also characterized by their small size, a property used to isolate hematopoietic stem cells (HSCs) in early studies (Jones et al., 1996; Radley et al., 1999). Additionally, a number of adult stem cell types are commonly described as “small cells” in the literature, such as the intestinal stem cells (Sato et al., 2011), regenerative cells of the fallopian tube (peg cells) (Paik et al., 2012), and stem cells in the rodent incisor (Biehs et al., 2013).

Intriguingly, the link between cell size and proliferative capacity may be evolutionarily conserved. In single-celled budding yeast, smaller cell size is associated with higher replicative capacity since genetic mutations that result in smaller yeast cells have longer replicative lifespans and vice versa (Yang et al., 2011). Similarly, in plants, apical meristem cells located in the shoot or root tips (comparable to stem cells in animals) are smaller in size compared to the more differentiated cells in the developing organs (Laufs et al., 1998; Jones et al., 2017).

On the other hand, senescent cells are usually associated with a large size (Muñoz-Espín and Serrano, 2014). In budding yeast, cells grow >50% larger in size across a replicative lifespan and enter senescence at a relatively constant size (∼10 μm) regardless of initial sizes (Mortimer and Johnston, 1958; Yang et al., 2011; Wright et al., 2013). A recent report on mouse HSCs suggested a causal link between cell size and stemness, whereby the enlargement of HSCs reduces their stem cell potential (Lengefeld et al., 2021). These authors also found that HSCs in both mice and humans increase in size during organismal aging, and the prevention of this age-dependent enlargement improves HSC function. Other recent studies examined molecular changes in large cells and proposed that enlarged cell sizes may induce senescence rather than simply being a phenotypic hallmark of senescence (Neurohr et al., 2019; Cheng et al., 2021; Lanz et al., 2021). For example, two independent papers reported upregulation of certain ER and lysosome-resident proteins in large mammalian cells (Cheng et al., 2021; Lanz et al., 2021), which has been known to induce a senescent phenotype (Lee et al., 2006; Ziegler et al., 2021).

Interestingly, the function of mTORC1, a major regulator of cell size and growth, was shown to be necessary for senescence (Demidenko et al., 2009; Laberge et al., 2015; Park et al., 2020). Aside from being a central regulator of cell growth, the mTORC1 pathway is also one of the key regulators of organismal aging. In fact, mTOR remains one of the few evolutionarily conserved pathways whose inhibition was found to extend the lifespan of all model organisms tested thus far, including yeast (Kaeberlein et al., 2005), *C. elegans (* Vellai et al., 2003 *)*, *Drosophila* (Kapahi et al., 2004), zebrafish (Khor et al., 2019), and mouse (Harrison et al., 2009). Together, these studies pose an interesting association between cell size and animal lifespan as both have links to the mTOR pathway. Indeed, two recent studies have revealed striking inverse correlations between cell size and animal lifespan in multicellular organisms. One study examined the size of adult pancreatic acinar cells across 24 mammalian species and identified a negative correlation between acinar cell size and animal lifespan (Figure 3C), independent of animal body size or metabolic rate (Anzi et al., 2018). Another study demonstrated that larger nucleoli are implicated with accelerated aging in worms, mice, and humans (Tiku et al., 2017). In addition, p16^INK4a^/CDK4 may be another pathway that regulates both cell size and animal longevity. p16 is a major driver of senescence (Coppé et al., 2011). In mice, aging is reversed by the clearance of p16 expressing cells and accelerated by the overexpression of p16 (Boquoi et al., 2015; Baker et al., 2016) as well as by mutation of multiple Rb phosphorylation sites (Jiang et al., 2022). p16 may also regulate cell size through its canonical function as an inhibitor of CDK4/6 (Tan et al., 2021). However, the correlation between cell size and longevity do not necessarily imply causation or directionality, and future work should therefore investigate the mechanistic drivers that link the two processes.

### Organ size control by cellular hypertrophy and atrophy

Growth (hypertrophy) and shrinkage (atrophy) in cell size is a common strategy of organ size regulation. Even in adulthood, tissue and organ sizes are dynamically controlled in response to the fluctuating environment. A larger organ size is generally associated with higher functional capacity. Many organs such as the liver, pancreas, kidney, lung, heart, and skeletal muscle undergo compensatory growth when experiencing increased functional demand (Goss, 1965). The liver, for example, can regenerate after partial hepatectomy by cell hypertrophy and increased polyploidy when cell division is inhibited (Diril et al., 2012). Pregnant mothers (of rodents and humans) that face elevated metabolic demand also experience significant cell hypertrophy in the liver (Milona et al., 2010) and pancreas (Rieck et al., 2009; Rieck and Kaestner, 2010). For cell types with limited proliferative capability, cell hypertrophy and atrophy can be readily used to regulate organ size. For example, the adult kidney has a low regenerative capacity. Human subjects or animals that have lost one kidney (e.g., for a kidney donation) experience rapid growth of the remaining kidney mostly through an increase in cell size (Johnson and Vera Roman, 1966) (Figure 3D). Even more surprisingly, transplanting an oversized kidney into a small-sized recipient (e.g., from adult to infants) results in regression of the transplanted kidney to match the size of the recipient (Churchill et al., 1991; Salvatierra et al., 1998), a process possibly accomplished through atrophy in cell size.

Growth and regression in organ size by changes in cell size maintains the overall organ structure and is likely more energy efficient than drastic alterations in cell number. The advantage of cell hypertrophy over hyperplasia can be important for tissues that undergo repeated cycles of growth and regression, such as the circadian/daily and circannual/seasonal cycles. Murine hepatocytes, for example, can experience daily oscillations in size of over 30% between dawn and dusk (Figure 3E) (Sinturel et al., 2017). In seasonal animals (e.g., seasonal breeders and hibernators), many organs undergo dramatic changes in size (Hindle et al., 2015) and cell types including adipocytes and different endocrine cells have been reported to display seasonal cell size variations (Klonisch et al., 2006; Wood et al., 2015). The mechanisms that account for cell division-independent control of cell size remain to be explored but will almost certainly depend on catabolic processes such as proteasome-mediated protein degradation (Acebron et al., 2014; Liu et al., 2021; Liu et al., 2022) and the manifold forms of autophagy (Miettinen and Björklund, 2015; Orhon et al., 2016).

## Perspectives on future cell size research

The past decade has seen rapid advances in mammalian cell size research, much of which was motivated by the original evidence for the cell size checkpoint first published in 1965 by Killander and Zetterberg. Over recent years, a number of independent labs have developed new methods to accurately and efficiently measure cell size (Cadart et al., 2018; Godin et al., 2010; Kafri et al., 2013; Liu et al., 2020; Serrano-Mislata et al., 2015; Son et al., 2012; Varsano et al., 2017). These efforts have delivered evidence that not only confirms the original seminal observation (Killander and Zetterberg, 1965a), but have further shown that size homeostasis in animal cells arise from regulation of both the duration and rate of cellular growth (Cadart et al., 2018; Ginzberg et al., 2018; Liu et al., 2022). Initial insights into the signal transduction pathways that underlie mammalian cell size checkpoint (Liu et al., 2018; Zatulovskiy et al., 2020) and target size specification (Tan et al., 2021) have begun to emerge.

Despite recent progress, our understanding of cell size control *in vivo* remains limited. In this review, we discussed the physiological importance of cell size. To date, quantification of cell size in tissues remains limited. With advances in 3D live-cell and deep tissue imaging, we expect more reports on measurements of cell size *in vivo* as well as in 3D organoid cultures. We envision that careful measurements of cell size in different tissue types and in different mutant contexts will reveal further interesting links between cell size and function, and shed light on how cell growth and size regulate and are regulated by animal physiology.
